# Contributing Factors Affecting the Severity of Metro Escalator Injuries in the Guangzhou Metro, China

**DOI:** 10.3390/ijerph18020651

**Published:** 2021-01-14

**Authors:** Hongwei Li, Yuxi Wang, Yingying Xing, Xiaochen Zhao, Ke Wang

**Affiliations:** 1 College of Civil and Transportation Engineering, Hohai University, Nanjing 210024, China; lihongwei@hhu.edu.cn; 2 CCCC Third Harbor Consultants Co., Ltd., Shanghai 200032, China; wangyx@theidi.com; 3 Key Laboratory of Road and Traffic Engineering of the State Ministry of Education, College of Transportation Engineering, Tongji University, Shanghai 201804, China; yingying199004@tongji.edu.cn (Y.X.); zhao_xiaochen@tongji.edu.cn (X.Z.)

**Keywords:** metro escalator accident, injury severity, Haddon matrix, risk factors

## Abstract

Urban rail transit has become one of the indispensable modes of public transportation in large cities. Escalators are ubiquitous in metro stations, as passengers typically use escalators when entering or leaving a metro station. Thus, escalators have become an accident-prone location. To develop suitable prevention strategies, it is necessary to understand the risk factors that affect the severity of escalator accidents. This study analyzed 967 escalator passenger accidents that occurred in the Guangzhou Metro from 2013 to 2015. The Haddon matrix was used to evaluate the interaction of humans, escalators, and environmental factors before, during, and after accidents. Then, the contributing factors associated with the severity levels were determined based on chi-square tests. Passengers aged 66 years and older are more vulnerable to serious injuries (*p* < 0.001), and previous health conditions are significantly related to the severity of the passenger’s injuries (*p* = 0.002). The weather conditions (rainy days) are also significantly related to the severity of escalator accident injuries (*p* = 0.039), and injured people with head injuries are at greater risk of being severely injured (*p* < 0.001). The analysis results of these risk factors can provide theoretical support for the metro operators to develop reasonable and effective preventive measures to reduce the escalator risk.

## 1. Introduction

With rapid development of economy and urbanization in China, metro has become an efficient, convenient, environment-protected, and sustainable traffic pattern to alleviate traffic jams. Up to 31 September 2018, 36 cities in China have been reported to operate metro with a mileage of more than 5000 km [1]. However, as more and more new lines are brought into service, the safety and reliability of metro operation has become an issue that is of great concern to the public.

The metro system includes underground, at grade, and elevated sections. Passengers usually take escalators as their priority to enter or leave a metro station [2]. As escalators are ubiquitous in metro stations, it is not surprised that escalators have become an accident-prone location. According to the survey, escalator-related injuries account for 67% of all the passenger injury accidents in Guangzhou Metro [3]. In other cities, serious escalator accidents in metro stations also happened. For example, on 5 July 2011, the equipment on the escalator of the Zoo Station on the Beijing Metro Line 4 was faulty, the elevator that was going up suddenly reversed and fell, the accident caused one death, two serious injuries and 28 minor injuries [4]. Another case related to metro escalator injuries took place on 2 April 2014 at the Jing’an Temple Station in Shanghai Metro, an escalator was retrograde during the morning rush hour. In this accident, 12 people were slightly injured, and one person was seriously injured [5].

To reduce the probabilities of escalator-related injuries, several studies has been conducted to investigate the epidemiology and cause of escalator-related injuries [6,7,8,9,10,11,12,13,14,15,16]. However, most of them focused on commercial escalators and only a few studies analyzed the accidents of metro escalator or heavy-duty escalator [2,3,17,18], which is designed specifically for transit system usage and substantially different from commercial escalators in terms of structure, configuration and performance [19]. Chi et al. [2] conducted an in-depth study of 194 escalator accidents in 2000 at Taipei high-capacity Metro Rapid Transit stations. The results show that most escalator accidents are caused by passengers performing other tasks. Li et al. [18] studied the causes of escalator injuries in Guangzhou South Railway Station, and found that escalator injuries occurred more frequently during periods that high-speed trains arrived intensively, and escalator injuries mainly happened in the lower middle of the upward escalator. Liu et al. [17] found that escalator injuries are more likely to occur in winter, second in summer. Xing et al. [3] analyzed the risk factors of different crowds for metro escalator accidents, and the results show that people aged 18–39 are more likely to suffer from escalator injuries when they are not accompanied, elder passengers are more likely to be injured because gradual decline of mental and physical capacities are found among the aging. More recently, Wang et al. (2020) identified the critical hazards affecting cascading escalator accidents through combining of FTDM (five-step task-driven method) and complex network analysis method [20]. Xing et al. (2020) analyzed risk factors affecting escalator-related injuries in metro stations based on Bayesian network and found that failing to stand firm, carrying out other tasks, not holding the handrail and another passenger’s movement are most influential factors resulting in escalator injuries [21]. Although useful and revealing, few of them have quantified the severity of the metro escalator accident and analyzed its influencing factors. Therefore, it is necessary to develop a paradigm to understand the complex interplay of the factors resulting in escalator accidents in metro stations.

The Haddon matrix has broadly been used in injury prevention since 1970 [22,23], as it provides a way to think about accidents thoroughly by diving an accident into three periods of time: pre-incident, incident, and post-incident. For example, Eddleston et al. [24] used the Haddon matrix to identify factors that increase the risk of fatal pesticide self-poisoning in Sri Lanka. The Haddon matrix was applied to identify contributing factors for tractor fatalities in Kentucky and wrong-way crashes on freeways in Illinois [25,26]. More recently, Rustagi et al. [23] applied the Haddon matrix to evaluate epidemiological risk factors of road traffic injury victims in Delhi, India. Yan and Yu [27] used the Haddon matrix to explore the medical response strategies to subway bombings.

Based on above considerations, there are at least two main purposes of the present study. The first one is to examine the contributing factors affecting the severity of metro escalator injuries. The second one is to provides a logical framework for understanding metro escalator injury causation. Therefore, this study analyzed 967 metro escalator passenger accidents in Guangzhou Metro stations in China from 2014 to 2016. Haddon matrix and statistical analysis method were used to explore the potential risk factors affecting the severity of passenger accident involving metro escalators. This result can be used to develop injury prevention measures in different phase to improve the safety of metro escalators.

## 2. Data

The metro escalator passenger accident data were obtained from the Guangzhou Metro Corporation (GMC). A total of 967 metro escalator accidents were recorded in 149 metro stations on 10 lines of the Guangzhou Metro from 2014 to 2016. The GMC prepared a case report for each incident and recorded it in their management information system (MIS). Each accident report records the details of the accident, including the date and time of the accident, the location, the age and gender of the passenger, the escalator number, the direction and speed of the escalator, the main cause of the accident, a detailed description of the accident, and any other factors considered to be relevant.

## 3. Methods

The Haddon matrix has been widely used to prevent collision injuries and to interfere with road safety. In addition, previous studies have shown that the Haddon matrix is an effective method in identifying and evaluating contributing factors for road traffic crashes [23,28]. In addition, the Haddon matrix has been also used to assess Kentucky tractor fatality risk factors [25], snow sports injury contributing factors [29], etc. There are mainly three steps using Haddon matrix [30]:

- The first step is to clearly identify the problem to be addressed using appropriate data and the problem in this paper is injury severity of escalator accidents.
- Second, one needs to define each row and column of the matrix. The columns of matrix are defined as the targets of change (host/human, agent/vehicle, environment), while the rows of matrix are defined by delineating the precise event and phases of change (pre-event, event, post-event). In this paper, the risk factors are derived from incident records from GMC and divided based on the definition of columns and rows of Haddon matrix.
- Once both dimensions of the matrix have been carefully defined, individual or group brainstorming is useful to generate ideas about interventions in each of the cells. Appropriate statistical methods could be used to explore the relationship between the problem and cells of matrix, which could help to propose useful interventions.

Therefore, factors affecting escalator injury severity were classified in 3 groups of human-, equipment-, and environment-related (Table 1).

The severity of injury was calculated using injury severity score (ISS). Those injuries described as having no visible scars in the accident record are considered possible injury, injuries with an ISS of 1–5 is classified as a minor injury, 6–15 is considered a moderate injury, 16–25 is severe, and over 25 is critical [31]. Since there are no serious or critical injuries in the accident report, the severity of the injury in this study includes only the following three categories: possible injury, minor injury, and moderate injury.

## 4. Results

### 4.1. Human Characteristics

Table 2 shows frequency distribution of the injury severity on the human factor. Fall is the main hazard pattern, accounting for 90.0% of moderate injuries, 91.5% of minor injuries and 100% of possible injuries. It can be seen in Table 2 that the proportion of female passengers among the passengers with moderate injuries in escalator accidents (60.0%) is higher than that of male passengers, and among the passengers with minor injuries, there are approximately twice as many female passengers (66.5%) as male passengers (33.5%).

Passenger’s age has a significant effect on the severity of the injury (*p* < 0.001). The mean age of the injured passengers was 52.7 years, ranging from 1 month to 92 years, and elderly people (aged 66 years and above) suffer more moderate injuries (52.0%).

The pre-existing health conditions of the passengers are related to the severity of the escalator injury (*p* = 0.002). The physical conditions of the passenger are rarely recorded; only 26 reports mentioned that the passenger was intoxicated, only 24 reports mentioned passengers suddenly feeling dizzy for their own reasons, for example, not eating breakfast causing hypoglycemia and dizziness, and only three reports mentioned that the passengers were disabled. Due to fewer passengers with health problems, 94.6% of escalator injuries were to passengers whose pre-existing health conditions were good. Although the escalator injury accidents involving unhealthy passengers only account for 5.2% of all accidents, 16.0% of the moderate injuries were to unhealthy passengers, indicating that unhealthy passengers are more likely to be involved in a moderate injury.

Although passenger behavior is not associated with the injury severity (0.752), it is the main reason causing escalator injuries. Of 967 cases, 856 escalator-related injuries (88.5%) are mainly caused by passengers’ risky behaviors, such as failing to stand firm (30.5%), performing other tasks (22.4%), not holding the handrail (11.3%) and so on.

Injured body regions are significantly related to the severity of the injury (*p* < 0.001). The head and neck (42.0%) and extremities (42.0%) are the most frequently injured body regions of the moderate injuries, and most of the moderately injured passengers are comatose or fractured after falling. The lumbar spine is also a common body region in escalator accidents, accounting for 16% of all moderate injuries.

### 4.2. Equipment Characteristics

The relationship between the injury severity and the equipment factors is displayed in Table 3. The escalator type is related to the severity of the injury (*p* = 0.016). From Table 3, it can be seen that with an increase in the severity of escalator injury, the proportions of possible injury, minor injury and moderate injury that happened on long escalators are reduced, indicating that accidents that occurred on ordinary escalators tend to result in more severe injuries than those on long escalators. Of all the moderate injuries in this study, the great majority of them happened on ordinary escalators (86.0%). This result probably occurs because passengers may be more careful and are more likely to hold the handrail and stand firm when riding a long escalator. In addition, it should be noted that the proportion of the long escalator-related injuries (20.3%) of all the escalator-related injuries was much higher than its proportion on all the escalators (5.8%), indicating that long escalators have a higher risk of escalator accidents than ordinary escalators.

Most commonly reported escalator injuries occurred during the upward movement of the escalator. This is simply because most of the escalators in Guangzhou Metro are going upward [3]. As a result, more accidents occurred in escalators that run upwards, so more serious injuries were also caused.

Although escalator service time is not significantly related to the severity of the accident, it can be seen that with the increase of escalator’s service life, the probability of minor and moderate injuries increases. Additionally, a large proportion of moderate injuries (88%) occurred on escalators with a service life of 10 years and above, while over 50% of minor injuries happened on escalators with a service life of 15 years and above. This result suggests that accidents occurring on escalators with a longer service life are likely to result in escalator injuries. There are two main reasons for this result. First, escalators are intended to age over time and more likely to fail. Second, those escalators with longer service time are usually from early metro lines, which have relatively large and continual passenger volume. As a result, escalators bear heavy load chronically, which accelerates their aging.

The escalator’s running speed of the escalator is not significantly related to the severity of the accident (*p* = 0.318), but it can be seen in Table 3 that almost half of the moderate injury accidents (48.0%) occur on escalators with a running speed of 0.65 m/s (2.34 km/h), indicating that high-speed escalators are more dangerous to passengers.

### 4.3. Environmental Characteristics

Table 4 reports frequency distribution of the injury severity on the environmental factors. Through a statistical analysis of escalator accidents that occurred in each metro station, 43.54% of the metro escalator passenger accidents occur in interchange stations, while interchange stations account for 14.7% of all metro stations [32]. This indicates that the probability of an escalator accident at the interchange station is greater. However, for moderate injuries there is no significant difference in the number of occurrences at non-interchange stations and at interchange stations. The results of the chi-square test also show that the type of metro station is independent of the severity of the injury (*p* = 0.952).

For the passenger flow of stations where escalator accidents occurred, 42% of the moderate injuries occurred in stations with a passenger flow below 50,000. As the passenger flow increases, the number of moderate injuries does not increase. The results of the chi-square test also show that the passenger flow is not significantly related to the severity of the accident (*p* = 0.059). Most of moderate accidents occur on working days (80.0%) and during working hours (68.0%), which indicates that the passenger flow is not related to the severity of the metro escalator accident.

The weather condition is a significant factor affecting the injury severity of metro escalator accidents (*p* = 0.039). Possible (64.9%) or minor injuries (61.7%) are more likely to occur on sunny and cloudy days, while moderate injuries occur more frequently on rainy days (56.0%).

## 5. Discussion

### 5.1. Risk Factors Affecting the Injury Severity of Escalator Accidents

**Figure 1:**
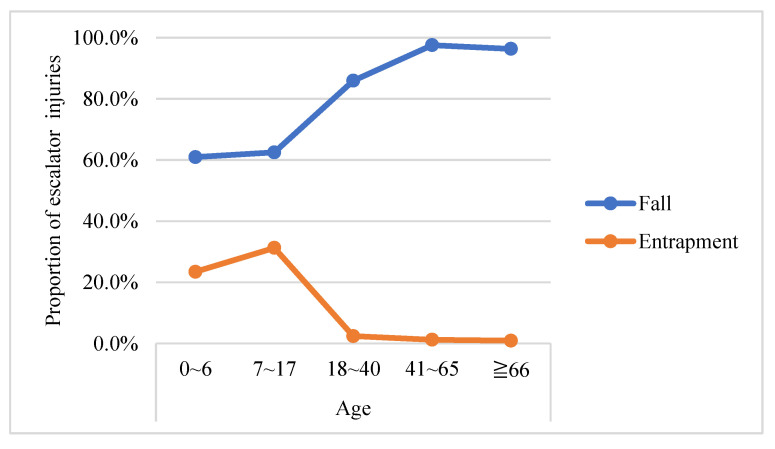
Proportion of escalator injuries categorized by age group and hazard pattern.

In terms of human factors, the frequency distribution of the severity of escalator accidents on various factors showed that among the passengers of moderate injuries in escalator accidents, the following groups were relatively large: female passengers, passengers aged 66 and above, and passengers of pre-health problems. This is probably because women are more likely to wear high-heel shoes, resulting in a higher probability to loss their balance and fall when taking the escalator [3], while the bodily and mental state of the elderly gradually decrease with age [33]. Fall is absolutely the main hazard pattern of escalator accidents, which is consistent with McCann and Zaleski’s [12] research conclusions. Failing to stand firm (28.0%), carrying out other tasks (18.0%), and other passenger’s movement (18.0%) are the main cause of moderate injuries. This result is consistent with the finding of Chi et al. [2]. After an in-depth analysis, it is found that moderate injuries caused by failing to stand firm mainly occur on elderly passengers, accounting for 78.6%. In addition, elderly passengers (62.5%) are more likely to be injured seriously than other age groups (37.5%) if they carry out other tasks when riding the escalator, indicating that elderly passengers are very vulnerable to escalator accidents and should be paid close attention to. Similar results are observed in Shanghai Metro that elderly passengers aged 60 and above account for 67.3% of all escalator-related injuries [34]. There are two main reasons for these results. First, people’s physical abilities will gradually decline with age, resulting in a higher probability to lose balance and fall when riding an escalator. As shown in Figure 1, there was a consistent increase with age in the proportion of injuries that are caused by falls, while there was a consistent decrease in the proportion of injuries that were attributed to entrapment by age group. This indicates that middle-aged (41–65 years) and elderly passengers (aged 66 years and above) are more likely to fall at an escalator, while children and teenagers are easier to be entrapped in the space between escalator steps or the escalator step and the sidewall. The other one is that elderly passengers are more likely to bring handbags or handcarts [35], which further increases the probability to fall.

For the escalator itself, the frequency of moderate injury occurring on ordinary escalators are higher than that of long escalators. However, considering the number of long escalators, long escalators are still a potential risk factor. Most of moderate injury occurred on escalators traveling upward (76%). This result may occur because, to control the flow in the metro station, more escalators in the Guangzhou Metro run upwards [36]. Although the running speed of the escalator is not significantly related to the severity of the accident, it is still a potential risk factor for escalator safety as over half of escalator-related accidents (54.0%) happen on high-speed escalators (0.65 m/s). This result is in line with previous finding that the escalator speed is a potential risk factor for escalator-related injuries [37]. Code for design of metro [38] stipulates that the rated speed of the escalator of the metro station should be greater than or equal to 0.50 m/s. At present, the escalators of the Guangzhou Metro adopt a rated speed of 0.65 m/s when the escalator load rate exceeds 50%. However, it may be more difficult for passengers, especially elderly people, children and passengers carrying out other tasks, to step on the pedals with the higher escalator’s running speed. Although only two moderate injuries are caused by the escalator failure in Guangzhou Metro, it should be noted that the escalator failure, such as sudden stop or reversal of direction, usually results in massive and severe injuries. For example, five people were injured due to escalator reverse malfunction in Ningbo Subway in 2016 [16].

**Figure 2:**
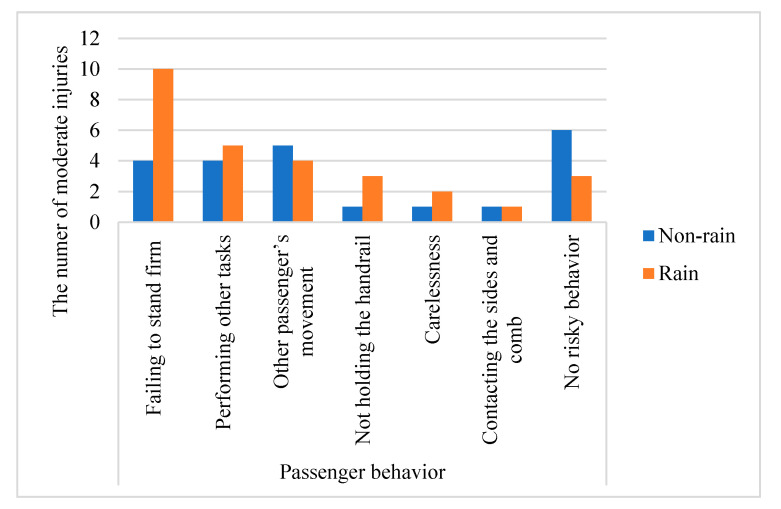
The number of moderate injuries caused by risky behaviors on non-rainy and rainy days.

For environmental factors, there is no significant difference in the frequency and probability of moderate injury between the interchange station and the non-interchange station. The chi-square test results show that passenger flow is not related to the severity of injury. In Xing et al.’s [3] research, it is also mentioned that a large passenger flow is not a potential risk factor for metro escalator accidents. There are more frequent moderate injuries at 9:30–17:29, but there is no significant difference in the severity of accidents at various time periods. For weather condition, passengers are more likely to be involved in moderate injuries on rainy days. This is probably because although most of the metro escalators are indoors, the water droplets on the passenger’s feet and on the umbrellas make escalators’ cover plates and stairs more slippery, which is more likely to cause a fall. After deep analysis of the data, it is found that 10 of 14 moderate injury accidents caused by failing to stand firm happened on rainy days (see Figure 2).

To further explore risk factors affecting injury severity of escalator accidents, a stepwise multiple linear regression analysis was performed using SPSS Statistics 22. (IBM, Armonk, United states) Six of 16 risk factors are founded to be significantly associated with injury severity of escalator accidents at 95% confidence level, i.e., hazard pattern, age, with company or not, injured body regions, long escalator, and rainy days, as shown in Table 5. The negative coefficient of hazard pattern indicates that fall is more likely to result in moderate injury, while the positive coefficient of age shows that the injury severity increases with age. It is interesting to find that passengers with company are more likely to be involved in more severe injuries. One possible explanation for this is that some accidental injuries may occur between accompanied passengers, such as crush injury. As expected, the positive coefficient of injury body region shows that head injury is usually more severe than other body regions. On the contrary, long escalator is found that has a negative impact on injury severity of escalator accidents. Compared with ordinary escalators, escalator injures happened on long escalators tend to be less severe. This is probably because passengers are more cautious when taking a long escalator. Rainy days is also a significant factor affecting injury severity and its positive coefficient indicates that passengers are more likely to be involved in moderate injuries on rainy days. These results confirm the importance of these factors, and thus should be paid more attention to improve escalator safety.

### 5.2. Associations between Levels of Factors Affecting Moderate Escalator Injuries

To further explore the factors affecting moderate escalator injury, associations among all contributing factors were revealed by the Cramer’s V analysis as illustrated in Table 6. Age is associated with four contributing factors, i.e., gender, with company or not, running speed, and traveling direction. Table 7 shows the distribution of those factors by age groups. It is interesting to find that male passengers aged 66 years and above have a greater tendency to be involved in moderate injuries than their gender counterpart (male: 60.0% vs. female: 46.7%), while the total proportion of female passengers with moderate injuries (60.0%) is higher than that of male passengers (40%). This is probably because elderly women are more cautious than elderly men when taking an escalator. Additionally, elderly female passengers are less likely to wear high-heel shoes than young women. Unlike other age groups, passengers aged 18–39 years are more likely to be involved in moderate injuries when they are without company. The association between age and running speed shows that passengers aged 66 years and above are more likely to be involved in moderate injuries (70.8%) when riding high-speed escalators (0.65 m/s). This result is intuitive as the higher the running speed of an escalator is, the more difficult it is for elder passengers to handle. Additionally, passengers aged 66 years and above are more likely to be involved in moderate injuries on upward escalators, while children (0–6 years) are prone to be moderately injured on downward escalators.

**Figure 3:**
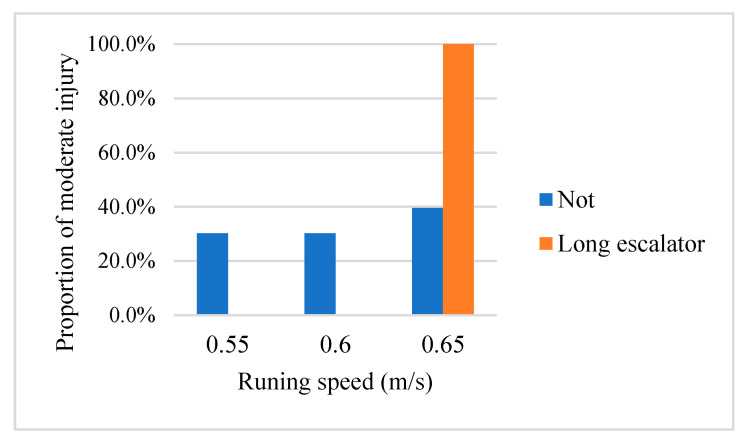
The proportion of moderate injuries categories by running speed and escalator type.

**Figure 4:**
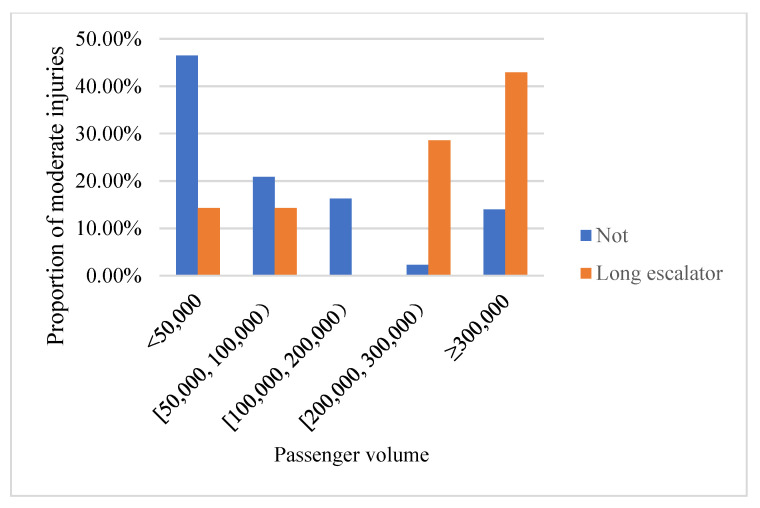
The proportion of moderate injuries categories by passenger volume and escalator type.

Although long escalator and running speed are not associated with injury severity of escalator accidents, there is a higher probability to result in moderate injuries on long and high-speed escalators. As shown in Figure 3, if a moderate injury happened on a long escalator, this escalator’s running speed is 0.65 m/s. Similar pattern could also be observed on the association between long escalator and passenger volume. As shown in Figure 4, there is a higher probability to result in moderate injuries on long escalators during heavy passenger volume. Therefore, in metro stations with heavy passenger flow, long escalator may be a potential source of risk. This result is consistent with previous study [3]. On a long escalator, passengers are more likely to walk on a long escalator to save time, especially on rush hours. In such cases, if the long escalator is running at high speed, it is easier to result in escalator accidents.

## 6. Conclusions

This study used the Haddon matrix to evaluate metro escalator accidents to highlight the risk factors affecting the severity of metro escalator injuries and then analyzed the frequency distribution of moderate injuries in escalator accidents in the Guangzhou Metro Station. Finally, the main factors affecting the severity of metro escalator accidents were studied. By using the Haddon matrix of the metro escalator accidents, one can then design interventions at different stages for different factors.

Before the accident occurred, metro operators could take effective measures to prevent escalator accidents. First, appropriate guidance to elevator should be provided to reduce escalator-related injuries of special passengers, including elderly passengers, disabled passengers, and pregnant women. Second, the preventive measures of regular patrol and periodic device test should be strengthened to ensure escalators’ safety status, especially escalators with long service time. Third, for long escalators, special emergency plan should be made and exercised regularly, especially in stations with heavy passenger volume. Additionally, station cleaners should remove excess water in a timely manner and keep the escalator steps dry. Meanwhile, anti-slip escalator cover plates and stairs could be adopted to improve the non-skid property of escalators. For passengers, there are also some tips that protect themselves from being hurt. Older passengers should not bring handbags or handcarts when riding an escalator since it appears to be associated with an increased risk of falling. Female passengers should be more careful than male passengers and try not to wear high-heel shoes while taking the escalator.

When an accident occurs, the passenger or staff should immediately press the emergency stop button of the escalator. Since passengers with head injuries are more susceptible to moderate injuries, the passenger should protect their head to prevent serious injury. Although the running speed of the escalator is not significantly related to the severity of the accident, it is still a potential risk factor for escalator safety as over half of escalator-related accidents (54.0%) happen on high-speed escalators (0.65 m/s). It is difficult for passengers, especially elderly people, children and passengers carrying out other tasks, to step on the pedals with the higher escalator’s running speed. Therefore, the speed of the escalator could be appropriately slowed within the standard operating speed range of the escalator during working hours.

After the accident, the first contact with the passengers is the frontline staff of the metro. Their assistance methods for escalator passengers also play a key role in the later treatment. Therefore, providing training in first-aid knowledge to the frontline staff is critical. When an escalator accident occurs, the staff can provide initial appropriate assistance to injured passengers to reduce the risk of further injury. In addition, the staff should promptly evacuate onlookers without affecting the ambulance’s treatment of the passengers. For the escalator, the staff should immediately overhaul the escalator and eliminate the fault to mitigate the impact on subsequent operations.

It should be acknowledged that this study has some limitations. Although accident data collected by Guangzhou Metro contain a lot of information, there is still a lack of useful information., such as the clothing and shoes of injured passengers (slippers, shopping bags, and footwear), using of cellphones while taking the escalator, any medical treatment, and so on. Therefore, it is suggested that future escalator accidents records could contain this information. In addition, the combination of escalator accidents records and surveillance video data, which provides more detailed information on escalator-related injuries, could be considered in future study.

## Figures and Tables

**Table 1:** Effective risk factors of escalator accidents based on the Haddon matrix.

Phases	Human	Equipment	Environment
Pre-incident	Age Gender Health condition With company or not	Escalator category Escalator’s service life	Station type Weather conditions
During incident	Passenger’s behavior Hazard pattern Injured body region	Traveling direction Escalator’s running speed Escalator safety status	Passenger flow Incident time
Post-incident	Whether to claim or not	Impact on the escalator	Pre-hospital care Ambulance service

**Table 2:** Frequency distribution of the injury severity on the human factor.

Human Factor	Categories	Injury Severity Level			Total Injury	*p*
		Possible Injury	Minor Injury	Moderate Injury		
Hazard patterns	Fall	37(100.0%)	805(91.5%)	45(90.0%)	887(91.7%)	0.847
	Entrapment	0(0.0%)	29(3.3%)	2(4.0%)	31(3.2%)	
	Scratch	0(0.0%)	9(1.0%)	1(2.0%)	10(1.0%)	
	Injuries caused by falling objects	0(0.0%)	35(4.0%)	2(4.0%)	37(3.8%)	
	Others	0(0.0%)	2(0.2%)	0(0.0%)	2(0.2%)	
Gender	Male	15(40.5%)	295(33.5%)	20(40.0%)	330(34.1%)	0.452
	Female	22(59.5%)	585(66.5%)	30(60.0%)	637(65.9%)	
Age	0–6	0(0.0%)	61(6.9%)	3(6.0%)	64(6.6%)	<0.001
	7–17	0(0.0%)	14(1.6%)	2(4.0%)	16(1.7%)	
	18–40	9(24.3%)	170(19.3%)	9(22.0%)	188(19.4%)	
	41–65	2(5.4%)	241(52.0%)	10(20.0%)	253(26.2%)	
	≥66	26(70.3%)	394(44.8%)	26(52.0%)	446(46.1%)	
Health condition	Healthy	34(91.9%)	839(95.3%)	42(84.0%)	915(94.6%)	0.002
	Unhealthy	3(8.1%)	41(4.7%)	8(16.0%)	52(5.4%)	
With company or not	NO	18(48.6%)	316(35.9%)	17(34.0%)	352(36.4%)	0.449
	Yes	19(51.4%)	552(62.7%)	33(66.0%)	604(62.5%)	
	Unknown	0(0.0%)	12(1.4%)	0(0.0%)	12(1.2%)	
Passenger behavior	Failing to stand firm	9(24.3%)	272(30.9%)	14(28.0%)	295(30.5%)	0.752
	Performing other tasks	10(27.0%)	198(22.5%)	9(18.0%)	217(22.4%)	
	Other passenger movement	6(16.2%)	157(17.8%)	9(18.0%)	172(17.8%)	
	Not holding the handrail	4(10.8%)	101(11.5%)	4(8.0%)	109(11.3%)	
	Carelessness	3(8.1%)	29(2.3%)	3(6.0%)	35(3.6%)	
	Contacting the sides and comb	0(0.0%)	11(1.3%)	2(4.0%)	13(1.3%)	
	Rushing for trains	0(0.0%)	8(0.9%)	0(0.0%)	8(0.8%)	
	Going in the wrong direction	0(0.0%)	7(0.8%)	0(0.0%)	7(0.7%)	
	No risky behavior	5(13.5%)	97(11.0%)	9(18.0%)	111(11.5%)	
Injured body regions	Head and neck	0(0.0%)	20(2.3%)	21(42.0%)	41(4.2%)	<0.001
	Face	0(0.0%)	196(22.3%)	0(0.0%)	196(20.3%)	
	Abdominal organs and lumbar spine	0(0.0%)	63(7.2%)	8(16.0%)	71	
	Extremities and pelvic girdle	0(0.0%)	440(50.0%)	21(42.0%)	461(47.7%)	
	External	0(0.0%)	143(32.7%)	0(0.0%)	143(14.8%)	
	Unidentified and unknown	37(100.0%)	18(2.0%)	0(0.0%)	55(5.7%)	

**Table 3:** Frequency distribution of the injury severity on the equipment factors.

Variable	Categories	Injury Severity Level			Total Injury	*p*
		Possible Injury	Minor Injury	Moderate Injury		
Long escalator or not	No	23 (62.2%)	704 (80.0%)	43 (86.0%)	770 (79.6%)	0.016
	Yes	14 (37.8%)	176 (20.0%)	7 (14.0%)	197 (20.4%)	
Direction	Upward	30 (81.1%)	762 (86.6%)	38 (76.0%)	830 (85.8%)	0.160
	Downward	7 (18.9%)	112 (85.5%)	12 (24.0%)	131 (13.5%)	
	Unknown	0 (0.0%)	6 (0.7%)	0 (0.0%)	6 (0.6%)	
Escalator’s service life	<10	3 (8.1%)	171 (19.4%)	6 (12.0%)	180 (18.6%)	0.071
	(10,15)	17 (45.9%)	259 (29.4%)	20 (40.0%)	296 (30.6%)	
	>15	17 (45.9%)	450 (51.1%)	24 (48.0%)	491 (50.8%)	
Escalator’s running speed (m/s)	0.55	7 (18.9%)	139 (15.8%)	13 (26.0%)	159 (16.4%)	0.318
	0.60	8 (21.6%)	263 (29.9%)	13 (26.0%)	284 (29.4%)	
	0.65	22 (59.5%)	478 (54.3%)	24 (48.0%)	524 (54.2%)	
Escalator safety status	Yes	1 (2.7%)	23 (2.6%)	2 (4.0%)	26 (2.7%)	0.783
	No	36 (97.3%)	857 (97.4%)	48 (96.0%)	941 (97.3%)	

**Table 4:** Frequency distribution of the injury severity on the environmental factors.

Variable	Categories	Injury Severity Level			Total Injury	*p*
		Possible Injury	Minor Injury	Moderate Injury		
Station Type	Non-interchange station	20(54.1%)	498(56.6%)	28(56.0%)	546(56.5%)	0.952
	Interchange stations	17(45.9%)	382(43.4%)	22(44.0%)	421(43.5%)	
Passenger Flow (PF)	<50,000	16(43.2%)	421(47.8%)	21(42.0%)	458(47.4%)	0.059
	50,000 ≤ PF < 100,000	5(13.5%)	133(15.1%)	10(20.0%)	148(15.3%)	
	100,000 ≤ PF < 200,000	7(18.9%)	121(13.8%)	7(14.0%)	135(14.0%)	
	200,000 ≤PF < 300,000	9(24.3%)	129(14.7%)	3(6.0%)	141(14.6%)	
	≥300,000	0(0.0%)	76(8.6%)	9(19.0%)	85(8.8%)	
Weather condition	Non-raining	24(64.9%)	543(61.7%)	22(44.0%)	589(60.9%)	0.039
	Raining	13(35.1%)	337(38.3%)	28(56.0%)	378(39.1%)	
Time	Operation opening time–07:29	1(2.7%)	22(2.5%)	1(2.0%)	24(2.5%)	0.827
	7:30–9:29	1(2.7%)	75(8.5%)	5(10.0%)	81(8.4%)	
	9:30–17:29	22(59.5%)	568(64.5%)	34(68.0%)	624(64.5%)	
	17:30–19:29	4(10.8%)	79(9.0%)	4(8.0%)	87()9.0%	
	19:30–operation closing time	9(24.3%)	136(15.5%)	6(12.0%)	151(15.6%)	

**Table 5:** Factors associated with injury severity of escalator accidents.

Variables	Coefficients	Std	*t*-Test	*p*-Value
Hazard pattern	−0.050	0.006	−8.143	0.000
Age	0.021	0.008	2.493	0.013
With company or not	10.045	0.019	12.429	0.015
Injured body regions	0.039	0.015	2.692	0.007
Long escalator	−0.054	0.022	−2.422	0.016
Rainy days	0.040	0.019	2.105	0.036

**Table 6:** Association between any two factors using Cramer’s V analysis.

Contributing Factors	Gender	Age	Healthy Condition	With Company or Not	Passenger Behavior	Hazard Pattern	Injured Body Region	Long Escalator or Not	Traveling Direction	Escalator’s Service Life	Running Speed	Escalator Safety Status	Station Type	Passenger Flow	Weather Condition
Age	0.445 *														
Healthy condition	0.089	0.286													
With company or not	0.017	0.451 *	0.147												
Passenger behavior	0.498	0.364	0.295	0.266											
Hazard pattern	0.209	0.316	0.214	0.375	0.341										
Injured body region	0.224	0.304	0.107	0.095	0.384	0.354 *									
Long escalator or not	0.212	0.325	0.138	0.075	0.361	0.231	0.152								
Traveling direction	0.172	0.707 *	0.118	0.289	0.496	0.184	0.307	0.043							
Escalator’s service life	0.397	0.374	0.176	0.209	0.316	0.326	0.397	0.342	0.309						
Running speed	0.261	0.376 *	0.017	0.153	0.270	0.174	0.154	0.420 *	0.096	0.264					
Escalator safety status	0.167	0.336	0.189	0.284	0.436	0.480 **	0.220	0.212	0.124	0.292	0.142				
Station type	0.230	0.304	0.053	0.044	0.385	0.240	0.405 *	0.223	0.068	0.447	0.205	0.025			
Passenger flow	0.302	0.262	0.142	0.215	0.331	0.302	0.380	0.499 *	0.215	0.376	0.296	0.396	0.858 **		
Weather condition	0.066	0.366	0.163	0.041	0.299	0.130	0.168	0.009	0.162	0.311	0.206	0.181	0.188	0.196	
Time of accident	0.288	0.236	0.221	0.321	0.332	0.189	0.296	0.391	0.309	0.348	0.273	0.140	0.139	0.272	0.181

* Significant at 0.05. ** Significant at 0.01.

**Table 7:** Distribution of contributing factors associated with age.

Contributing Factors		Age Group (Years)				
		0–6	7–17	18–39	40–65	≥66
Gender	Male	5.0%	10.0%	0.0%	25.0%	60.0%
	Female	6.7%	0.0%	30.0%	16.7%	46.7%
With company or not	Without company	5.9%	0.0%	41.2%	11.8%	41.2%
	With company	6.1%	6.1%	6.1%	24.2%	57.6%
Running speed (m/s)	0.55	15.4%	15.4%	23.1%	15.4%	30.8%
	0.60	7.7%	0.0%	23.1%	30.8%	38.5%
	0.65	0.0%	0.0%	12.5%	16.7%	70.8%
Traveling Direction	Upward	0.0%	5.3%	10.5%	18.4%	65.8%
	Downward	25.0%	0.0%	41.7%	25.0%	8.3%

## Data Availability

Restrictions apply to the availability of these data. Data was obtained from Guangzhou Metro and are available from the authors with the permission of Guangzhou Metro.
